# Caution

**Published:** 1879-08

**Authors:** 


*Caution.*—Prevent all unnecessary crowding of persons around the body, especially if in an apartment. Avoid rough usage and do not allow the body to remain on the back *unless the tongue is secured.*

Under no circumstances hold the body up by the feet or roll it over a barrel. On no account place the body in a warm bath.

